# Body donor reperfusion and re-ventilation in medical training: an Italian study testing SimLife®

**DOI:** 10.3389/fmed.2024.1488285

**Published:** 2025-01-23

**Authors:** Irene Neri, Giulio Vara, Antonietta Fazio, Maria Vittoria Marvi, Foteini-Dionysia Koufi, Elisa Boschetti, Simone Lodi, Giulia Adalgisa Mariani, Marilisa Quaranta, Anna Maria Billi, Alessandra Ruggeri, Carlo Barausse, Cyril Brèque, Annalisa Plava, Veronica Moretti, Lucia Manzoli, Stefano Ratti

**Affiliations:** ^1^Cellular Signalling Laboratory, Anatomy Center, Department of Biomedical and Neuromotor Sciences (DIBINEM), University of Bologna, Bologna, Italy; ^2^Institut PPRIME UPR 3346, Chasseneuil-du-Poitou, France; ^3^Department of Sociology and Business Law, University of Bologna, Bologna, Italy

**Keywords:** medical simulation, body donation, surgical training, technology innovation, SimLife® technology

## Abstract

**Background:**

Medical simulations have emerged as a valuable tool in anatomical-medical training, allowing healthcare professionals to gain hands-on experience in a controlled and safe environment. One such simulation platform is SimLife®, which uses the Pulse for Practice (P4P) system to enable realistic restoration of airflow (“re-ventilation”) and blood flow (“revascularization”) in bodies donated to science.

**Objective:**

This study aimed to evaluate the feasibility of introducing SimLife® technology in Italy. Additionally, it assessed the impact of this technology across various medical specialties, utilizing a minimal number of donated bodies.

**Methods:**

The study utilized the existing body donation program and dissection rooms at the Anatomy Center of the University of Bologna. 62 participants from 13 medical specialties performed simulations using the SimLife® P4P platform. Post-simulation, structured interviews were used to collect data on the interventions performed, participant perceptions of the technology’s usefulness, enjoyment, and willingness to repeat the experience, as well as critical issues encountered.

**Results:**

Key findings include that 86% of participants rated SimLife® technology as extremely useful for *post-lauream* training, while 84% found it highly beneficial for team-building activities. A total of 31 interventions were successfully performed across various anatomical regions, with participants reporting high satisfaction and a strong willingness to repeat the simulation experience.

**Conclusion:**

The findings support the effectiveness of SimLife® technology for body donor re-ventilation and revascularization, reinforcing its value for medical training across various specialties.

## Introduction

### Ethical foundations and importance of simulation in medical training

*Primum non nocere* (“first of all, do no harm”) is a fundamental principle in medical education, emphasizing the ethical responsibility of healthcare professionals to prioritize patient safety. Modern medical training recognizes the importance of not only minimizing physical harm but also fostering emotional and psychological well-being through compassionate care—a concept referred to as “pedagogy of kindness” (1, 2). This approach highlights the need for comprehensive training that integrates technical skills with empathetic, patient-centered care. However, traditional training models often lack the immersive and safe environments required to adequately prepare medical professionals.

### Challenges with existing models

The evolution of medical and surgical disciplines has exposed limitations in traditional training methods, such as the Halstedian apprenticeship model (“see one, do one, teach one”) (3). In this scenario, simulation models have proven to be valuable tools to improve and assist medical training (4, 5, 6, 7, 8). Synthetic simulators, while offering risk-free practice, frequently lack anatomical fidelity and are costly to produce and maintain (9, 10, 11). Organic simulators, such as animal models, face ethical restrictions and limited applicability due to anatomical differences (12). Human cadavers, though invaluable for understanding anatomy (13, 14, 15), fail to replicate dynamic physiological conditions like blood flow, bleeding, and ventilation, essential for developing advanced surgical skills (16, 17).

### SimLife® technology

To address these limitations, innovative solutions like SimLife® have emerged. SimLife® technology integrates “re-ventilation” (restoration of airflow to the lungs) and “revascularization” (restoration of blood flow through a simulated circulatory system) to dynamically animate bodies donated to science. These features enable simulations that closely mimic real-life surgical conditions, offering a unique blend of anatomical precision and physiological realism. Several studies have demonstrated the benefits of perfused human body models in enhancing realism in surgical training, improving skills acquisition, and trainee satisfaction (18–20, 21, 22). SimLife® builds on these advancements, addressing critical gaps in simulation fidelity, such as replicating dynamic physiological processes like blood flow and ventilation, and providing anatomically precise models for complex surgical procedures (23, 24, 25, 26).

### Study goals and broader significance

This study aimed to test the feasibility of introducing SimLife® technology in Italy and to evaluate its impact across multiple medical specialties while maximizing the use of body donors. By leveraging the body donation program at the Anatomy Center of the University of Bologna, this study explored SimLife®‘s potential for improving medical education, team training, and surgical skills acquisition. Beyond assessing feasibility, this research sought to underscore the broader implications of adopting dynamic simulation technologies. Indeed, SimLife® not only advances surgical training capabilities but also highlights the ethical significance of body donation, paving the way for transformative educational practices in Italy and globally.

## Materials and methods

### Study objectives

The present study aimed to evaluate the impact of SimLife® P4P technology on medical education and formation through a comprehensive investigation based on two hypotheses. The first hypothesis aimed to identify the medical fields where SimLife® technology could have the most significant impact. The second hypothesis focused on evaluating how SimLife® technology could enhance medical training and education. To address these hypotheses, a wide-spectrum simulation approach was employed, involving 62 medical and surgical participants from 13 different specialties. This approach was chosen to comprehensively evaluate the versatility and applicability of SimLife® technology across various medical fields. By testing the platform on diverse procedures and anatomical regions, we aimed to identify its strengths and limitations, ensuring its broad utility in medical training. At the end of the simulation, participants were asked to answer a structured interview to determine their anatomical districts of interest, and the different interventions performed during the SimLife® simulation. Interventions success rates were also assessed. Success rate was determined based on participants’ ability to successfully complete the intended surgical or medical procedures and measured through participant self-assessment. Participants were asked to self-evaluate the execution of procedural steps and the achievement of procedural objectives. Additionally, participants’ perception regarding the usefulness and impact of SimLife® technology for research, *pre-lauream* (*pre-graduate*) and *post-lauream* (*post-graduate*) training, and team-building were assessed through 6-points Likert-scale questions. Finally, the limitations and the general opinion on the simulation were examined through open-answer questions.

### Study setting

The Anatomy Centre of the University of Bologna provided the setting for this study, with two fully equipped dissection rooms and an established body donation program that supports both educational and research activities (27). This infrastructure was instrumental in organizing the simulation sessions detailed in this study. The Anatomy Centre hosts two fully equipped dissection rooms: the main dissection room, equipped with four workstations and an audio-video-endoscopic system for recording and streaming activities, and a high-technology anatomical room with a modular system for two surgical workstations. In 2023, the Anatomy Centre organized a total of 584 h of dissection room activities, including 250 h for *post-lauream* training, 224 h for *pre-lauream* training, and 115 h for advanced courses. These activities reflect the Centre’s commitment to advancing medical education and research through its body donation program. The dissection room activities for this study were supported by the Anatomy Centre’s Near-Peer Teaching (NPT) program, which involves senior medical students assisting in educational and training activities. This program, active since 2003, played a key role in facilitating the organization and execution of the simulations, leveraging its structured framework to support participants during the SimLife® sessions. Further details on the NPT program are available in Orsini et al. (28).

### Body donors’ preparation

Two bodies donated to science via the Anatomy Centre body donation program were defrosted three days prior the simulation in a room at 18°C. Age, sex, height, body type and cause of death of the two body donors are reported in Table 1.

**Table 1:** Profile of the body donors object of the SimLife® simulation.

	Body donor 1	Body donor 2
Age	80 y.o.	76 y.o.
Sex	Male	Male
Height	166 cm	172 cm
Body type	Obese	Overweight
Death cause	Chronic respiratory insufficiency	Cerebral ischemia

The day before the simulation, the body donors were prepared by Simedys specialized personnel according to a new configuration, different from that described by Delpech et al. (24), allowing many more surgical simulations to be carried out.

The preparation process involved the following steps:

1 Cannulation for vascular access:

- Right common carotid artery and right internal jugular vein: two arterial and two venous cannulas were placed. For each pair, one cannula was directed centrally toward the heart, and the other peripherally toward the head. A pressure sensor was positioned on the right to measure aortic pressure.
- Femoral arteries and veins: two arterial and two venous cannulas were inserted in each femoral vessel, with central and peripheral directions for each pair. For the right femoral vessel, cannulas were placed as low as possible to simulate extracorporeal membrane oxygenation (ECMO) or interventional radiology procedures.
- Brachial arteries and veins: two arterial and two venous cannulas were inserted into the brachial vessels, with central and peripheral directions for each pair.

2 Tracheotomy and re-ventilation:

- A tracheotomy was performed to facilitate re-ventilation of the lungs. Alternative intubation techniques were avoided due to the risk of accidental esophageal intubation, which could compromise the model.

3 Nasogastric tube insertion:

- A nasogastric tube was inserted to aspirate gastric fluids and prevent complications during simulations.

4 Vascular tree cleansing:

- The vascular system was flushed with 12 liters of water at 37°C using low pressure (0.4 bar or 300 mmHg).
- Alternating injections into arterial and venous cannulas ensured the removal of native blood and clots, leaving the system clear.

5 Connection to SimLife® module:

- On the day of the simulation, arterial and venous cannulas were connected to the Pulse for Practice (P4P) SimLife® Control module using 2–4 mm diameter pipes.
- A peristaltic pump (a pump that uses a rotating mechanism to compress and push fluid through flexible tubing) injected a water-based blood avatar heated to 37°C into the vascular system.
- Solenoid valves (electromechanically controlled devices used to regulate fluid flow) on arterial inputs created pulsatile flow to mimic heartbeats. These valves were synchronized using a programmable logic controller (PLC), a digital device designed to automate processes through precise timing and coordination.

**Figure 1:**
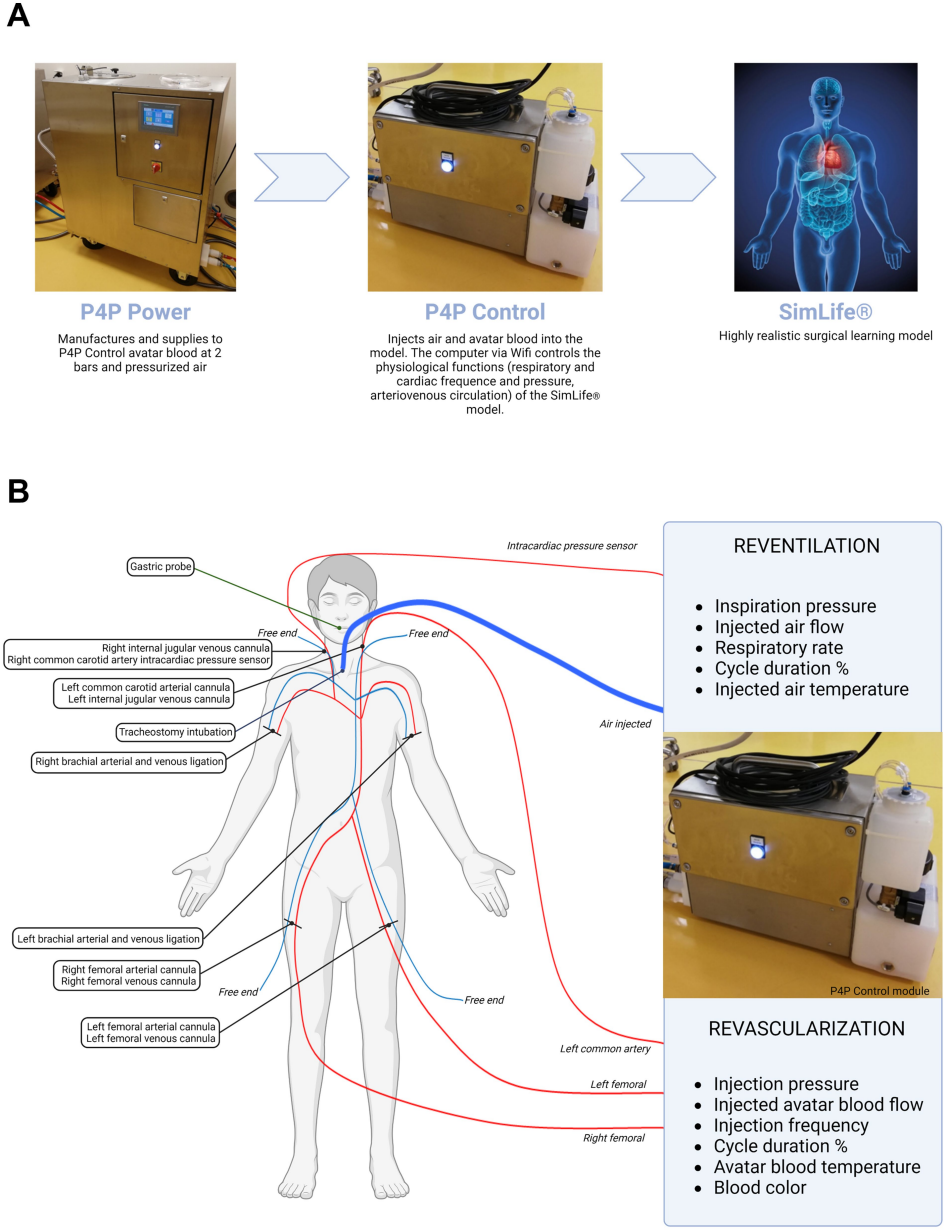
Schematic representation of P4P SimLife® technology. **(A)** Principle of action. **(B)** Vascular and aerial accesses and related connections to the P4P Control module. The principle of action of SimLife® technology has been described in detail by Delpech et al. (24).

A schematic representation of the P4P SimLife® technical module principle of action and of the vascular and aerial accesses are reported in Figure 1.

### Simulation process

Two bodies donated to science through the University of Bologna body donation program were defrosted and prepared for SimLife® simulation. Each body donor was placed in a separate dissection room and was connected to a distinct P4P SimLife® module 30 min before the start of the simulations. The functionality and performance of the SimLife® module were priorly presented to the teams in order to appreciate its capabilities. The presentation was held by SimLife® specialized personnel, who also answered any question asked by the teams. The activities were organized in two consecutive days in order to allow the participation of 62 medical professionals belonging to 13 different medical specialties and divided into 18 teams. Each team was given a total of 2 h to perform the simulation. The general information regarding the timetable of the activities is reported in Table 2. Detailed demographic information, including age, gender, and specialty distribution, is presented in the Results section under “Participant Demographics.”

**Table 2:** Timetable of the activities for the two days Siemedys SimLife® P4P simulation.

Day 1				Day 2			
Body donor 1		Body donor 2		Body donor 1		Body donor 2	
8 am–10 am	General surgery team	8 am–10 am	Otorhinolaryngology team	8 am–10 am	Neurosurgery team 2	8 am–10 am	Vascular surgery team
10 am–12 pm	Oral and Maxillofacial surgery team 1	10 am–12 pm	Neurosurgery team 1	10 am–12 pm	Cardiothoracic surgery team	10 am–12 pm	Orthopedic surgery team 2
12 pm–2 pm	Gynecology team 1	12 pm–2 pm	Oral and Maxillofacial surgery team 2	12 pm–2 pm	Orthoplastic surgery team	12 pm–2 pm	Orthopedic surgery team 3
2 pm–4 pm	Gynecology team 2	2 pm–4 pm	Orthopedic surgery team 1	2 pm–4 pm	Anesthesiology team	2 pm–4 pm	Urology team
4 pm–6 pm	Radiology team	4 pm–6 pm	Anatomists team and Anatomy tutors team				

### Data collection

After the 2-h simulation sessions, spread over 2 days, participants were interviewed. The structured interview consisted in 10 questions divided into 3 parts, respectively investigating the anatomical districts of interest and the intervention performed (part 1), the overall activity evaluation (part 2) and the general comments on the simulation (part 3). The questions’ structure was mixed, including open answer questions and 6-points Likert scale questions. The meaning of each point on the Likert scale was verbally explained to all participants to ensure a consistent understanding of the scale. This explanation was provided by the same facilitator for all participants prior to completing the survey, ensuring standardization across the study. The scheme of the structured interview is reported in Supplementary material 1.

### Data analysis

SPSS statistical package, version 25.0 (IBM Corp., Armonk, NY, USA) was used to statistically analyze the collected data. The Kruskal-Wallis test for independent samples was selected as a non-parametric alternative to ANOVA due to the small sample sizes and the non-normal distribution of the data, which was confirmed using the Kolmogorov–Smirnov test. Similarly, Fisher’s Exact Test was chosen for categorical data analysis because it is robust for small sample sizes, providing accurate results where chi-square tests may be unreliable. Only *p*-values <0.05 were considered statistically significant. Moreover, frequency was calculated for every answer. GraphPad Prism, version 8.0 (GraphPad Software, Boston, MA, USA) was used to graphically visualize the structured interviews answers. The open answers were transcribed *verbatim* by using the Nvivo12 software (QSR International, Melbourne, Australia), a computer-assisted qualitative analysis tool that allowed empirical material to be more easily organized and managed. The Nvivo12 software allows the analysis of unstructured or semi-structured data, such as interviews, enabling the coding and organization of information so that its content could be explored, and theories could be built and tested on the textual data. The descriptive data from the interviews were then analyzed using thematic analysis – in which common topics, ideas and patterns of meaning were identified in categories – to examine and understand the data at a general level (29). To ensure inter-coder reliability, two researchers independently coded a subset of the responses and compared their results. Discrepancies were resolved through discussion and consensus, and the final coding framework was applied to the entire dataset. The thematic analysis identified common topics, ideas, and patterns of meaning, which were categorized into macro-themes for a comprehensive understanding of the data.

## Results

### Participant demographics

A total of 62 participants, representing 13 medical specialties, participated in the study. The majority were specialists (71%), with the remaining 29% consisting of medical residents undergoing specialized training. The age distribution ranged from 19 to 44 years, with an average age of 40 years, reflecting the inclusion of both seasoned professionals and trainees. The demographic breakdown, including gender and specialty distribution, is summarized in Table 3.

**Table 3:** Demographics of the participants.

Demographics	Participants
Gender	
Female	19
Male	43
Age	
19–23	5
24–30	13
30+	44
Medical specialty	
Anatomist (Anatomy tutor)	13 (5)
Anesthesiology	3
Cardiothoracic surgery	3
General surgery	3
Gynecology	4
Interventional neuroradiology	3
Neurosurgery	3
Oral and maxillofacial surgery	7
Orthopedic surgery	8
Orthoplasty	2
Otorhinolaryngology	2
Urology	2
Vascular surgery	5
	Total participants = 62

### Anatomical districts of interest and interventions performed

**Figure 2:**
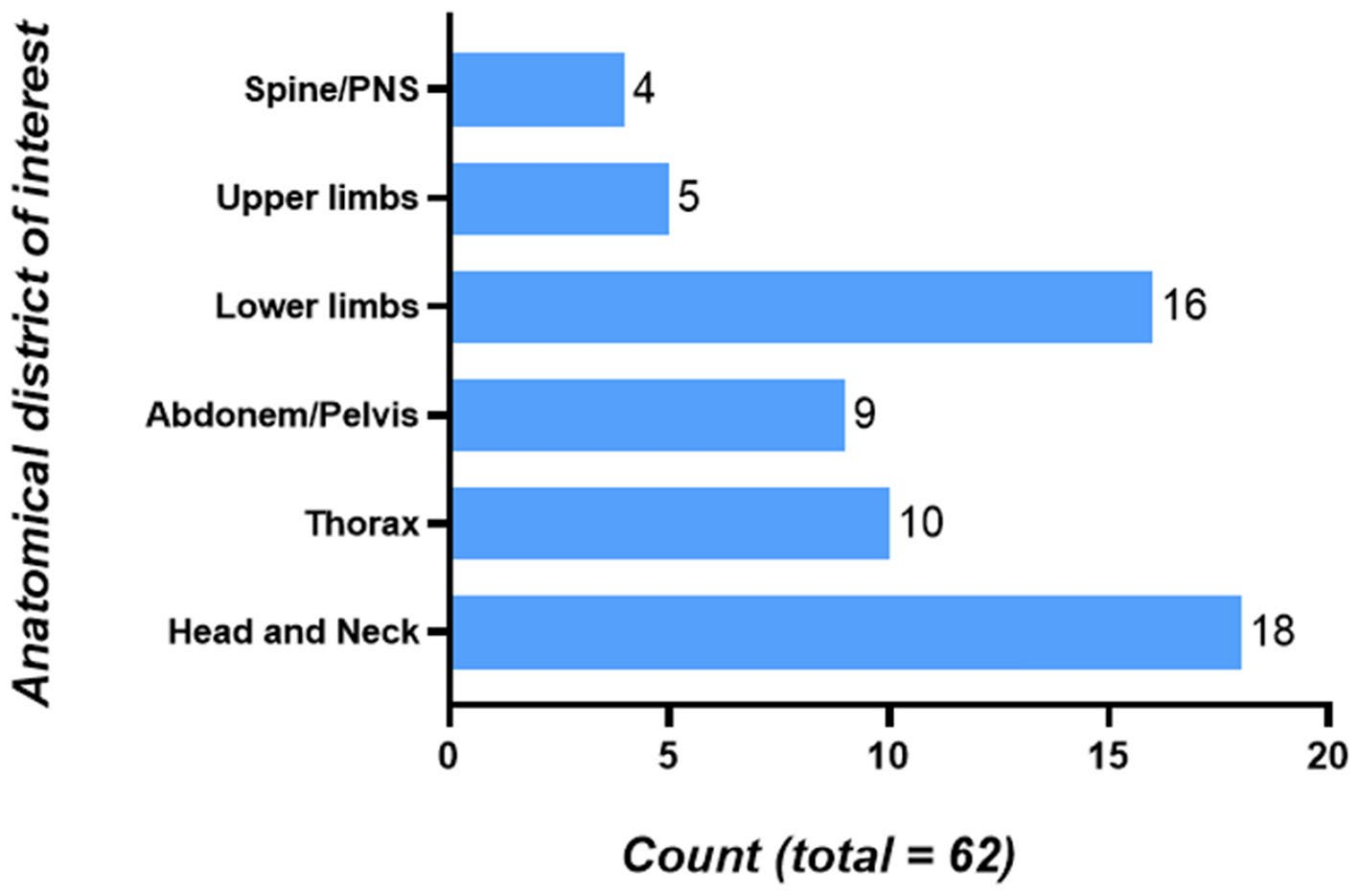
Anatomical districts of interest of the participants. 18 people were interested in the Head and Neck region; 10 people were interested in the Thorax region; 9 people were interested in the Abdomen/Pelvis region; 16 people were interested in the Lower limbs region; 5 people were interested in the Upper limbs region; 4 people were interested in the Spine/PNS region. PNS, Peripheral Nervous System.

The anatomical district of interest of every participant and the interventions performed in this SimLife® simulation were investigated through open-answer questions. Among the 62 participants, 29% declared that their anatomical district of interest was the head and neck region. Another 31% declared to be interested in the thorax/abdomen/pelvis region. The 26% declared to be interested in the lower limbs, while the 8% declared to be interested in the upper limbs. Finally, another 6% declared to be interested in the spine/peripheral nervous system (PNS) (Figure 2).

**Figure 3:**
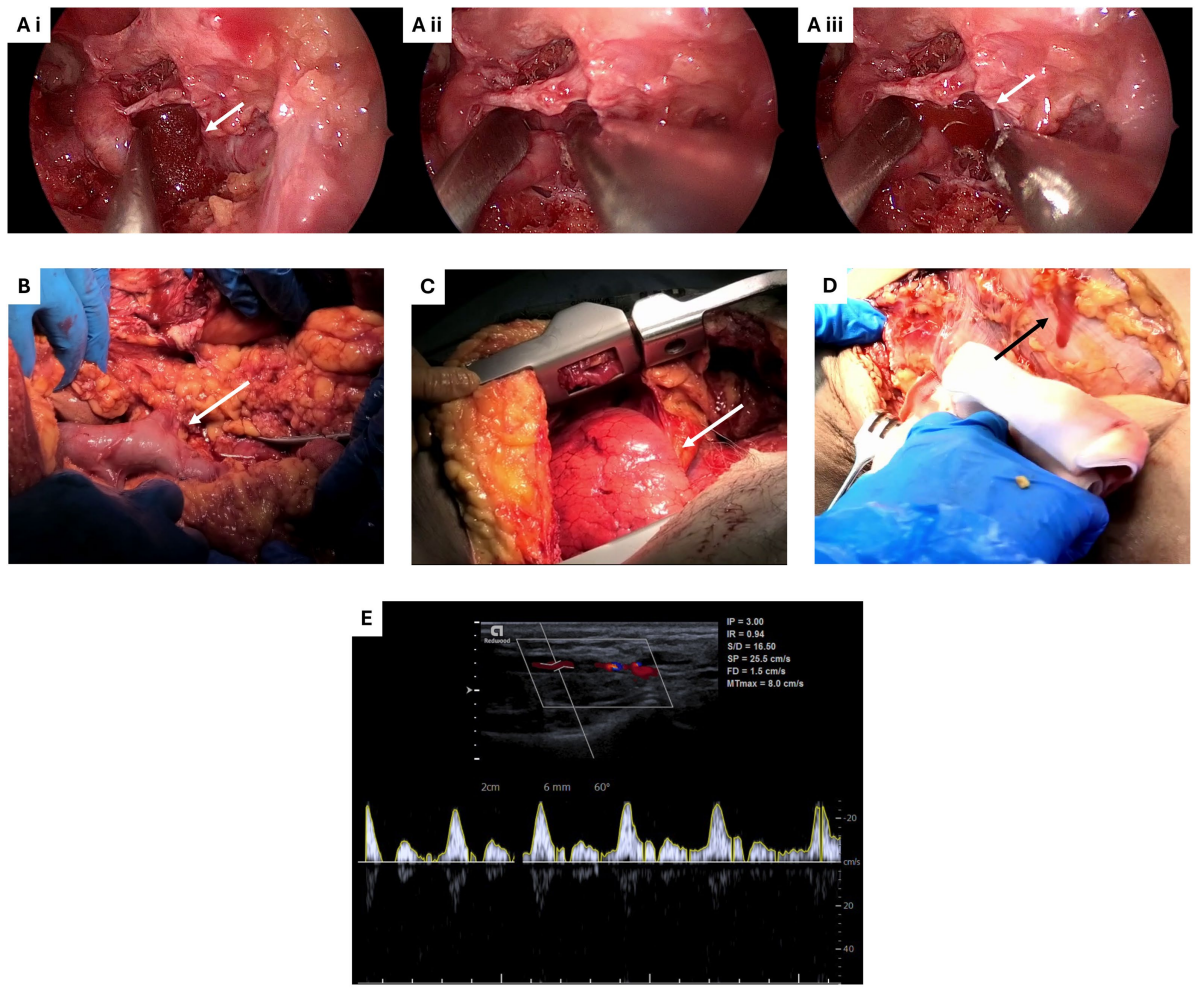
Exemplificative images of some of the surgical interventions performed during Simedys simulation. **(Ai–iii)** Endoscopic endonasal approach to skull base, highlighting the bleeding of the internal carotid artery. White arrows indicate the avatar blood. **(B)** Exposure of inferior vena cava, highlighting the turgidity of the vein due to reperfusion (white arrow). **(C)** Lungs asportation, highlighting the right lung expansion phase during re-ventilation (white arrow). **(D)** Knee dissection, highlighting the bleeding of superficial vessels (black arrow). **(E)** Pulsed wave doppler ultrasound of the radial artery. The image illustrates the velocity waveform obtained from the radial artery, demonstrating characteristic systolic peaks followed by diastolic flow, indicative of the efficacy of the simulated arterial blood flow. The scale on the right represents velocity (cm/s), and the time (sec) is shown on the horizontal axis.

Overall, a total of 31 different interventions were performed. These varied from explorative/dissecting interventions such as gallbladder exploration and ankle dissection to more complex surgical operations such as heart and lungs asportation and galeo-pericranial microvascular free flap. The complete list of the interventions performed during the two-days SimLife® simulation is reported in Table 4. Figure 3 reports exemplificative images of some of the surgical interventions performed. Moreover, shortcuts of the galeo-pericranial microvascular free flap intervention and of the knee medial and lateral ligament reconstruction are reported, respectively, as Supplementary Videos S1, S2. Finally, the success rate of the interventions was assessed. 100% of the participants declared that the intervention they performed was successful.

**Table 4:** Interventions performed during the SimLife® simulation.

Anatomical district	Interventions performed
Head and neck	Maxillary lateral sinus lift Endoscopic endonasal approach to skull base Middle ear endoscopy Parotidectomy Pterional craniotomy Transethmoidal approach to sella and parasellar area Galeo-pericranial microvascular free flap Neck dissection
Thorax/Abdomen/Pelvis	Laparoscopic sleeve gastrectomy Lobectomy Chest muscles dissection Heart and lungs asportation Right-hemisphere gastrectomy Bilateral radical nephrectomy with open anterior trans-peritoneal approach and bilateral subcostal incision Exposure of aorta and vena cava Lysis of intestinal and omental adhesions Exploration of gallbladder Intra-operative ICG fluoroangiopathy Bladder exposure
Upper limbs	Radial forearm flap Radial artery graft Pulsed wave doppler ultrasound of the radial artery Wrist dissection
Lower limbs	Femoral popliteal bypass Medial collateral ligament surgery Total hip arthroplasty with ilioinguinal approach Knee medial and lateral ligament reconstruction Ankle dissection Knee dissection
Spine/PNS	Anterior lumbar interbody fusion Vagous nerve exposure

### SimLife® simulation evaluation

**Figure 4:**
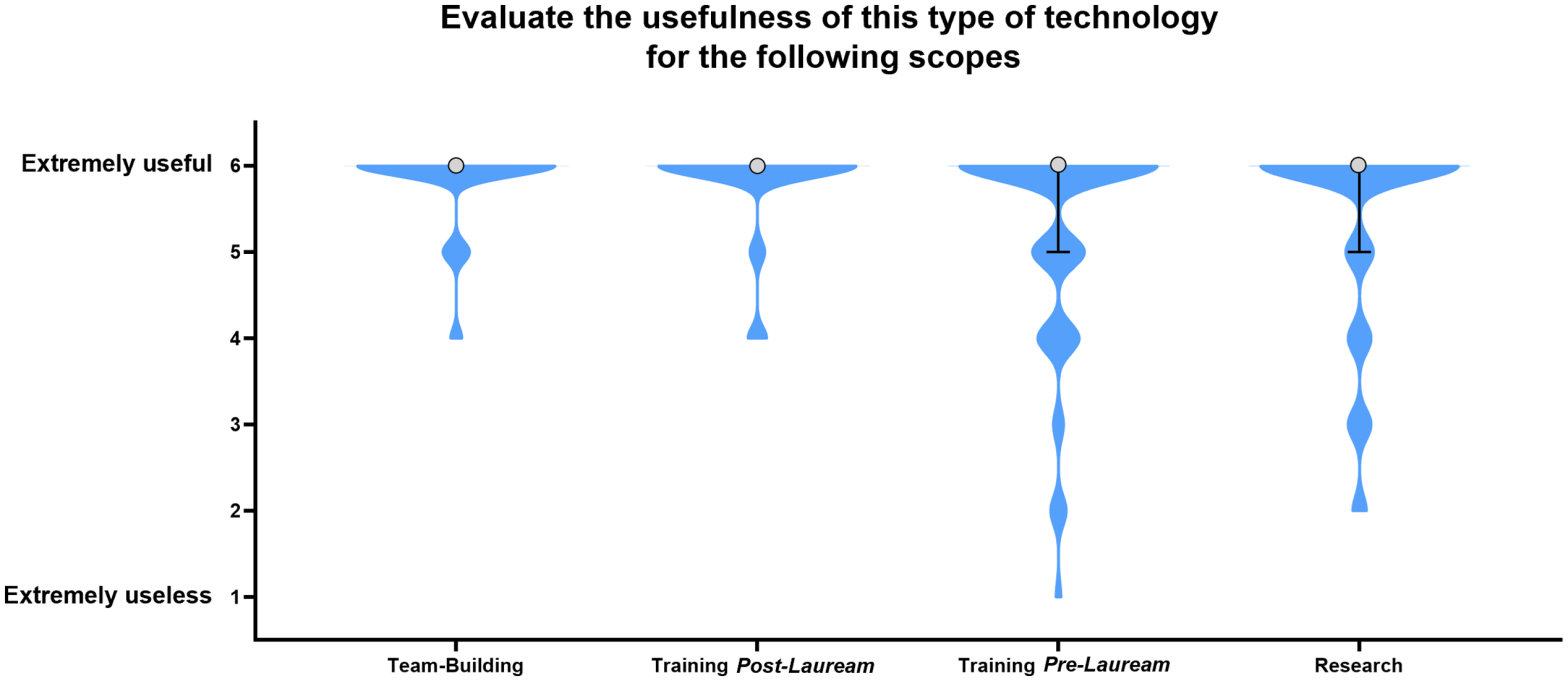
Evaluation of the usefulness of SimLife® technology for various applications: Team-Building, Training *Post-Lauream*, Training *Pre-Lauream*, and Research. Violin shape shows the distribution of participant responses. The circle indicates the median response value, and the error bars represent the interquartile range (IQR). Where error bars are not visible, the IQR = 0, indicating uniform participant responses for that category.

The activity evaluation, represented by the participants’ perception about the usefulness of SimLife® technology, the participants’ enjoyment/appreciation and the participants’ willingness of repeating the experience, was assessed using 6-points Likert-scale questions. Overall, the participants’ perception about the usefulness of SimLife® simulation was extremely positive. Out of the 62 responders, 69% affirmed that SimLife® technology is absolutely useful for research. The 61% affirmed that this technology is absolutely useful for *pre-lauream* training. The 86% declared that SimLife® technology is absolutely useful for *post-lauream* training. Finally, 84% of participants affirmed that this technology is absolutely useful for team-building. The median and the interquartile range (IQR) for each 6-points Likert-scale question on this section is reported in Figure 4.

**Figure 5:**
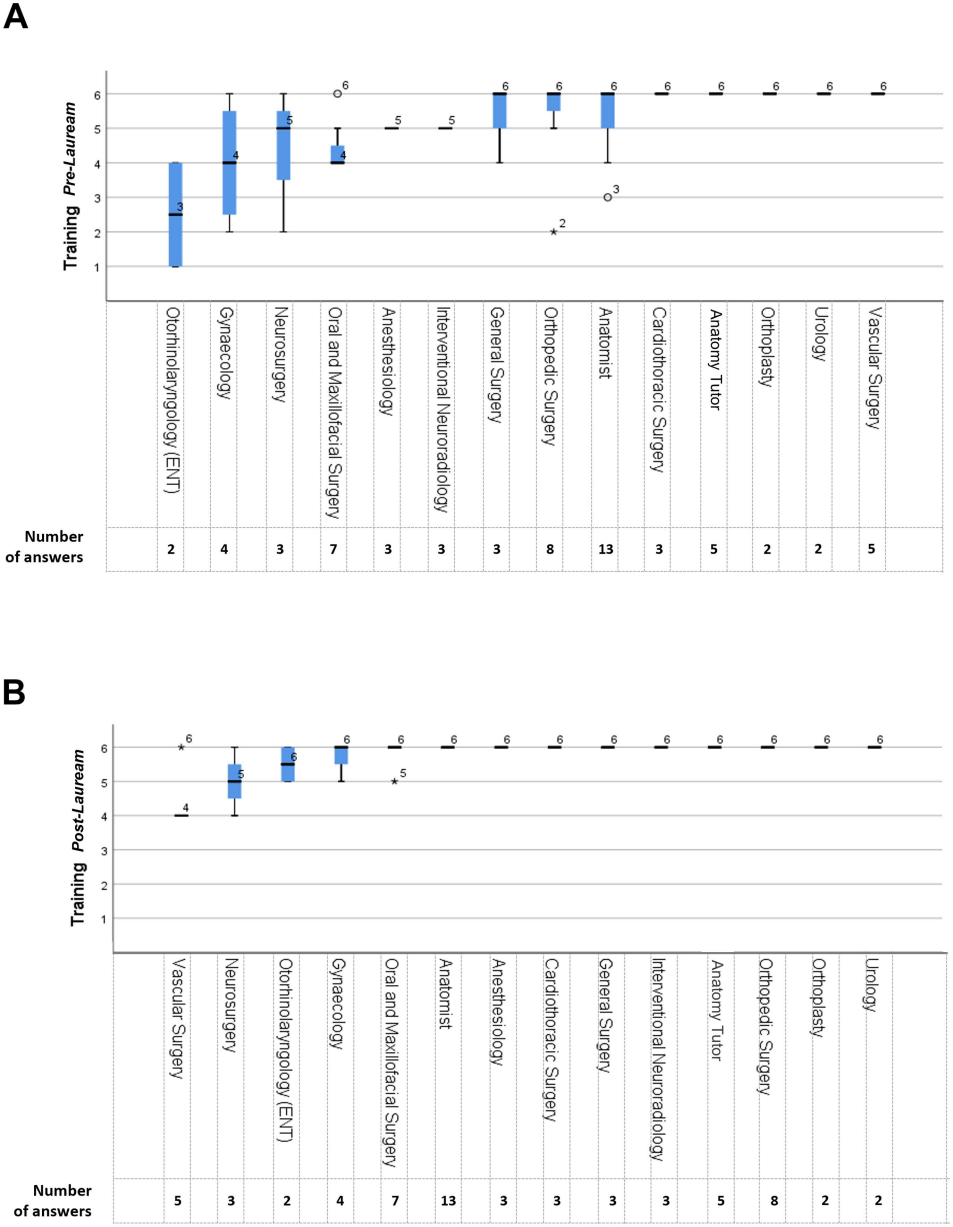
Perceived usefulness of SimLife® technology across medical specialties. **(A)** Perceived usefulness of SimLife® for *pre-lauream* training. **(B)** Perceived usefulness of SimLife® for *post-lauream* training. Y-axis indicates the perceived usefulness score (1 = Not useful at all, 6 = Extremely useful), while X-axis reports the different medical specialties. Each boxplot represents the interquartile range of responses, with the line indicating the median score and the whiskers showing the overall range. The circles indicate the mild outliers, representing responses that are outside the interquartile range, while the stars indicate the extreme outliers, representing responses well outside the typical range.

To determine if there were statistically significant differences in the perception of SimLife® technology’s usefulness across different medical specialties, the Kruskal-Wallis test was performed between the medical specialty indicated by the participants and the answers to the 6-Points Likert scale question “Evaluate the usefulness of this type of technology for the following scope: research, training *pre-lauream*, training *post-lauream*, team-building,” with 1 = not useful at all and 6 = extremely useful. The results were subsequently utilized to conduct pairwise comparisons between the different medical specialties. The application of the Kruskal-Wallis test revealed statistical significance in the variability of responses only for the *pre-lauream* training and *post-lauream* training categories. The graphical visualization of the results of the two tests are reported in Figures 5A,B, respectively. While the majority of medical specialties have reported a good or very good perception (mean score 5 and 6) of the SimLife® technology’s usefulness in both categories, some medical specialties reported a lower utility. In particular, regarding the *pre-lauream* training category, the otorhinolaryngology specialists expressed a less favorable opinion compared to other specialties (mean score 3). Concerning the pairwise comparison for this category, significant differences were highlighted between this specialty and several others. For example, otorhinolaryngology specialists have expressed a significant lower opinion compared to the anatomy specialists (*p* = 0.017), the vascular surgery specialists (*p* = 0.004), the urology specialists (*p* = 0.017), and the cardiothoracic surgery specialists (*p* = 0.009). Regarding the *post-lauream* training category instead, the vascular surgery specialists expressed a less favorable opinion compared to other specialties (mean score 4). Also in this case, the pairwise comparison for this category has highlighted significant differences with other medical specialties. For example, vascular surgery specialists have expressed a significant lower opinion compared to the maxillofacial surgery specialists (*p* = 0.001), the anatomy specialists (*p* < 0.001), and the cardiothoracic surgery specialists (*p* = 0.001).

**Figure 6:**
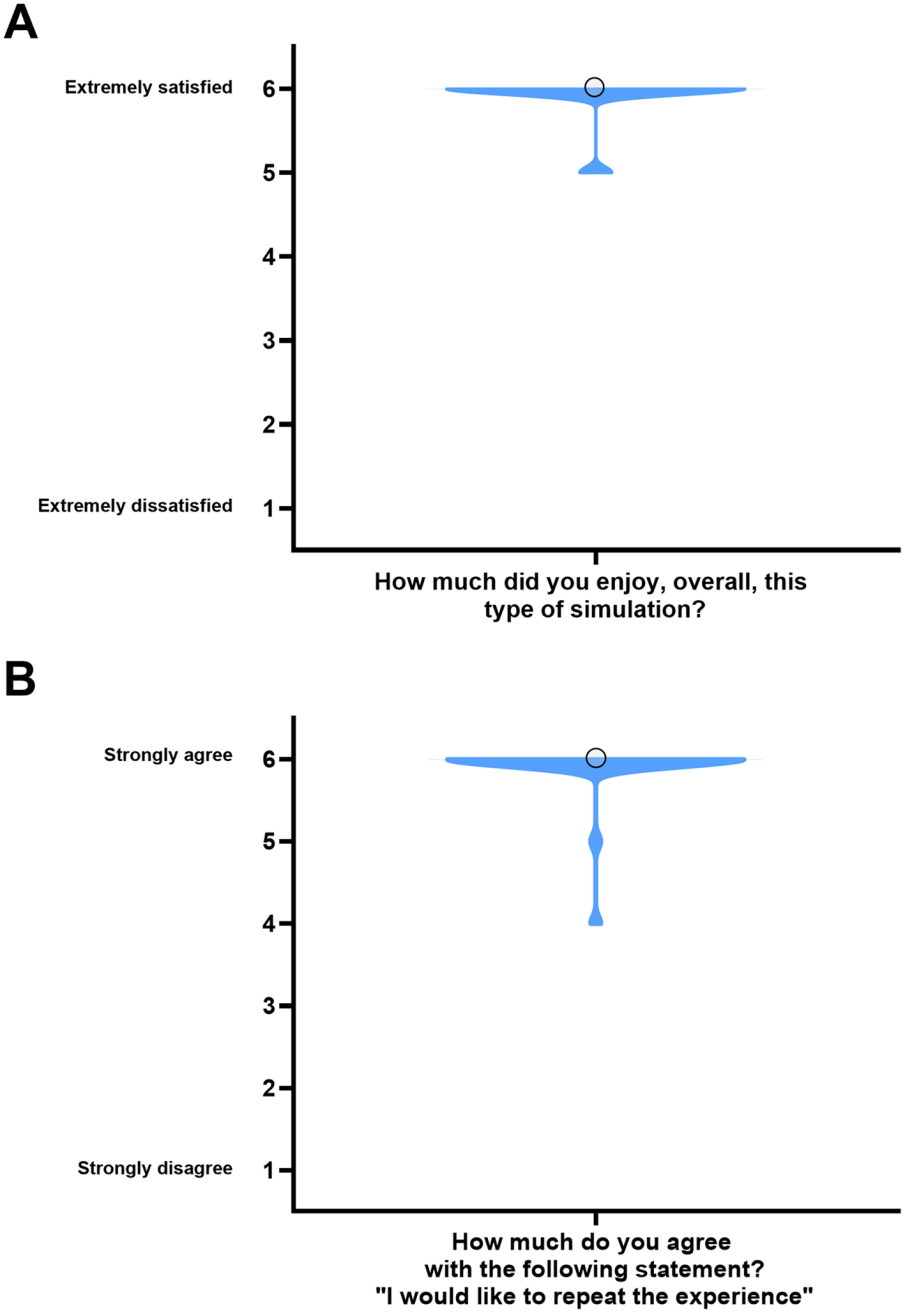
Participants’ evaluation of the simulation experience. **(A)** Participants’ overall satisfaction with the simulation. The violin plot represents the distribution of participants’ responses. The circle indicates the median score. **(B)** Participants’ willingness to repeat the experience. Violin shape shows the distribution of responses. The circle indicates the median score. For both panels, the IQR = 0 and is therefore not displayed.

The participants’ enjoyment/appreciation resulted to be very high. Indeed, 100% of the participants declared that they enjoyed the activity (97% absolutely yes and 3% yes). Moreover, 99% of the participants declared that they would like to repeat the SimLife® simulation (98% absolutely yes and 1% yes). The median and IQR for both of these two 6-points Likert-scale questions are reported in Figure 6.

To determine if there were statistically significant differences in the enjoyment of the simulation across different medical specialties, the Kruskal-Wallis test was performed between the medical specialty of the participants and the answers to the 6-Points Likert scale questions “How much did you enjoy, overall, this type of simulation” and “Would you like to repeat this experience,” respectively. However, this analysis did not provide statistically significant results (*p* = 0.734 and *p* = 0.556 respectively).

### Critical issues and general comments on the simulation

At the end of the interview, participants were asked to indicate any critical issue they encountered and to express any other comment they had. Regarding the question “Did you notice any critical issue in this type of simulation?,” 4 main themes emerged from the thematical analysis:

- Bleeding of small vessels: although SimLife® P4P system ability of recreating blood flow in the major vessels was highly appreciated, some concerns were raised about the small ones. Example: *«The system is very efficient in recreating the circulation of large vessels, but unfortunately not the small ones»*.
- Lack of coagulation: another issue that was reported was the lack of coagulation. Example: *«[One problem is the] lack of coagulation, which makes difficult to control bleeding»*.
- Body donor preparation: the length of the preparative phase and the vascular accesses were also addressed. Example: *«Vascular access technique and preparation time could be improved»*.
- Body donor position and characteristics: finally, the impossibility of moving the human bodies and the problems related to their characteristics were reported. Examples: *«Inability to obtain additional body positions»*; *«Difficulty with the obese subject»*.

To establish whether the observed problems encountered during the SimLife® simulations were randomly distributed among the various anatomical districts or whether there was a statistically significant correlation, the Fisher Exact test was performed between the anatomical district of interest indicated by participants and the question “Did you notice any critical issue in this type of simulation?.” The results indicate that there was no relation between the critical issues encountered during the simulation and the anatomical district of interest (*p* = 0.598).

Finally, the sentiment derived from the answers to the final open question (“Other comments”) was assessed. The general sentiment about the SimLife® P4P simulation was good, with 100% of the responders reporting a positive experience. Examples: *«[…] It proves to be a valid tool for simulations»*; *«Very useful for medical training»*; *«[…] I would recommend this kind of training to anyone in the medical field»*.

## Discussion

Medical simulations have revolutionized the landscape of medical education and training, offering a myriad of benefits to both aspiring healthcare professionals and seasoned practitioners (30). Traditionally, medical training relied heavily on observation and apprenticeship, where novice practitioners learned under the guidance of experienced mentors. While this approach is valuable, it can be limited by the unpredictability and the variability of real-life patient cases, and most importantly by patient safety concerns. To overcome the traditional training related problematics, the integration of simulation platforms has become increasingly crucial. Indeed, medical simulations have become an indispensable tool in medical formation due to their ability to create a safe, risk-free learning environment that grants medical students and professionals the skills, knowledge, and confidence necessary to deliver high-quality patient care (31, 32). One such cutting-edge simulation platform that has gained widespread recognition is SimLife®, a state-of-the-art system designed to provide realistic scenarios and immersive experiences to medical participants. In this study, the success of 31 interventions across various anatomical regions demonstrated the technology’s capacity to replicate real-life scenarios, offering a highly effective platform for surgical training and skill acquisition. The SimLife® platform is based on the dynamization of body donors by the pulsatile revascularization with simulated (“avatar”) blood warmed to 37°C and re-ventilation (24). In this way, SimLife® represents an innovative tool that allows the creation of a realistic, safe, and controlled environment by implementing a futuristic technology to bodies donated to science. The present study aimed to perform a wide-spectrum test of the SimLife® platform, involving several medical specialties. 62 medical professionals participated to this study. The simulation, that lasted two days, took place at the dissection rooms of the Anatomy Centre of the University of Bologna and involved 2 bodies donated to science that were each connected to a SimLife® P4P console. After the simulation, participants were interviewed to determine their anatomical district of interest and the interventions performed during the simulation. Moreover, all the participants were asked for their perception on the simulation. Finally, critical issues encountered during the simulation and the general sentiment of the experience were also assessed. 29% of the participants were interested in the head and neck region, while another 31% were interested in the thorax/abdomen/pelvis region. 26% declared interest in the lower limbs, while the upper limbs and the spine/peripheral nervous system (PNS) received, respectively, 8 and 6% of interest. The SimLife® simulation encompassed a total of 31 different interventions, ranging from simple explorative/dissecting procedures to more complex surgical operations. All the interventions were successful, underlining the efficiency of this simulation system even though the interventions were performed on a limited number of bodies donors. Moreover, this result is important because it underlines the possibility of using similar settings for team training. Team training refers to a structured process or program designed to enhance the effectiveness, collaboration, communication and performance of a group of individuals who work together as a team. The goal of team training is to improve communication, coordination, problem-solving, and overall team dynamics, leading to better outcomes and higher productivity. Indeed, team training is particularly important in the medical fields, and the value of simulations for enhancing the medical team training experiences is widely recognized (33). The participants’ perception of SimLife® simulation was extremely positive. According to the Likert-scale responses, 69% of the responders believed that SimLife® technology was absolutely useful for research, 61% for *pre-lauream* training, 86% for *post-lauream* training, and 84% for team-building purposes. The high percentages of positive responses demonstrate that SimLife® successfully addresses various educational and training requirements for medical professionals. To delve into the nuances of participants’ perceptions across different medical specialties, the Kruskal-Wallis test was employed. This analysis was pivotal in uncovering specific areas where the platform’s perceived utility varied, particularly in relation to *pre-lauream* and *post-lauream* training requirements. The Kruskal-Wallis analysis revealed significant differences in participant perceptions across specialties, providing critical insights into how SimLife® technology meets the unique needs of various fields. Otorhinolaryngology participants reported lower satisfaction, particularly regarding its utility for *pre-lauream* training. This likely reflects the high precision and tactile feedback demands of this specialty, where procedures often involve delicate structures and intricate techniques. Expanding SimLife® to include finer tactile tools or modules specifically designed to replicate small, delicate procedures could significantly enhance its value for otorhinolaryngology and similar specialties. Similarly, vascular surgery participants expressed lower satisfaction with *post-lauream* training, which may be attributed to the lack of microvascular simulation capabilities. Future advancements in SimLife® could incorporate models that mimic microvascular conditions, enabling high-fidelity simulations for specialties that demand precision at a microscopic level. These improvements would broaden the platform’s applicability and better address the diverse requirements of medical and surgical training. SimLife® demonstrated considerable potential for team-building activities, as 84% of participants stated to find this technology absolutely useful for team-building. Moreover, 100% of participants reported enjoyment and 98% expressed a willingness to repeat the simulation. Taken together, these findings highlight the platform’s ability to foster collaboration and enhance intra-team dynamics in a controlled environment. Regular team-building simulations using SimLife® could address real-world challenges, such as improving intra-team communication, coordination, and decision-making under pressure—skills that are critical during high-stakes medical procedures. By simulating realistic scenarios, SimLife® enables teams to practice resolving conflicts, adapting to unforeseen complications, and making collective decisions, ultimately translating these skills into clinical practice. The consistent positive feedback underscores the value of integrating team-based simulations into routine training programs to strengthen team cohesion and effectiveness. Indeed, these results are concordant with the other studies using this technology that are present in literature (34–37). Despite the overall positive sentiment emerged from the general comments on the simulation, participants did identify some critical issues. Through a thematic analysis, four main themes emerged:

- Bleeding of small vessels: While the system demonstrated excellent efficiency in recreating blood flow in major vessels, concerns were raised about the representation of smaller vessels. Indeed, for logistical reasons, Simedys personnel was unable to prepare the body donors as soon as they arrived at the Bologna Anatomy Center. The preparation of the human bodies shortly after death/thawing is strongly recommended for the successful outcome of the simulation and therefore this could have affected the blood flow in the small vessels. However, if delayed preparation eventually affects micro-vascularization is still to be investigated. Future versions of the technology could incorporate models that simulate small vessel bleeding using controlled flow mechanisms, enhancing the fidelity of surgical scenarios.
- Lack of coagulation: Participants pointed out the absence of coagulation, which made it challenging to control bleeding during certain scenarios. However, SimLife® simulation models are to be considered hemophilic patients and therefore require the usage of a mono or bipolar electric scalpel. Indeed, this requirement makes the interventions challenging as indicated by several participants. Developing clotting simulation models, such as using biochemical agents or programmable flow restrictors, could address this issue and expand the utility of SimLife® for hemostatic training.
- Body donor preparation: Some participants expressed concerns about the length of the preparative phase and the difficulty with vascular access. Improving the vascular access technique and minimizing preparation time could enhance the overall efficiency of the simulation. To achieve this, the programming of surgical simulations needs to be improved, and the simulated operating technique needs to be known in advance. Unfortunately, this step was not possible due to logistical reasons.
- Body donor position and characteristics: Participants highlighted the inability to move body donors due to connections to the P4P Control module and issues related to body characteristics, such as obesity. Similarly to what concerns the body donor preparations, to grant the best outcome of the simulation Simedys personnel should know the surgical technique object of the simulation in advance. Indeed, this knowledge allows to define the body donor’s operating position beforehand. However, due to logistical reasons and also to the wide-spectrum approach that was followed, this step was not possible. On the other hand, technological innovations such as lightweight or modular configurations for the P4P module, could allow for greater flexibility and a wider range of positional adjustments during simulations.

These findings collectively underscore the imperative for ongoing advancements to address the identified limitations, ultimately fostering a more comprehensive and realistic training environment for medical professionals. Nevertheless, participants consistently conveyed an overwhelmingly positive sentiment, with a unanimous 100% reporting a favorable experience. Respondents expressed the simulation’s utility as a valid tool for medical simulations, emphasizing its significance in medical training. While the positive feedback highlights the SimLife® P4P simulation’s strengths, the identified areas of improvement, including issues with small vessel representation, coagulation dynamics, body donor preparation, and adaptability to diverse body mass index, offer a constructive roadmap for refining and expanding the simulation’s capabilities in future iterations. Such a balanced evaluation acknowledges both the notable successes and the avenues for enhancement, contributing to the ongoing discourse on advancing medical simulation technologies for comprehensive training in the healthcare domain. Finally, given the results presented in this study, it is relevant to point out that SimLife® technology transcends its role as a very useful tool in medical training, embodying also a pivotal innovation that emphasizes the invaluable contribution of body donation to medical science. Indeed, by integrating realistic simulation environments with the anatomical precision provided by actual human bodies, SimLife® technology not only enhances the educational landscape for medical professionals but also serves as a profound tribute to the generosity of body donors. Several recent studies point out the ethical considerations that are part of implementing body donation to medical training, such as the respect and dignity owed to the body donors (the “silent teachers”) (38, 39) and the critical importance of body donations in advancing medical education and research (40, 41). The ethical dimensions of body donation remain a cornerstone of simulation-based medical training, providing a foundation for advancing both education and research. Technologies like SimLife® amplify the value of body donors by creating opportunities for dynamic and realistic training scenarios, ensuring that their contributions have maximum impact. This synergy between technological advancement and ethical considerations highlights how innovation can complement respect for donor dignity. As simulation technology evolves, ethical frameworks must adapt to ensure the continued dignity and recognition of body donors. Simulation platforms like SimLife® enhance this respect by transforming static anatomical models into dynamic, life-like systems, thereby elevating the educational and training value of donated bodies. Future advancements could include donor recognition programs or digital memorials that acknowledge the invaluable role of donors in advancing medical education, further integrating ethical considerations with technological progress.

## Conclusion

In conclusion, this comprehensive study aimed to assess the impact of SimLife® P4P technology on medical training and education in Italy. The wide-ranging simulation approach involving 62 medical and surgical participants from 13 specialties successfully identified the technology’s potential impact across diverse medical fields. The simulation, conducted at the Anatomy Center of the University of Bologna, showcased the overall efficiency of SimLife® for various medical fields. The simulations, encompassing 31 different interventions, were universally successful, underlining the system’s effectiveness and the potential for specialized training settings even with a limited number of body donors. Despite the overwhelmingly positive perceptions of participants, highlighted by Likert-scale responses indicating the utility of SimLife® technology for research, *pre-lauream*, *post-lauream* training, and team-building, our study has also highlighted notable variations in its perceived utility across the various medical specialties. These insights underscore the importance of tailoring the simulation experience to meet the distinct demands of each medical field. Indeed, addressing the specific requirements identified could be pivotal in refining the SimLife® platform. Moreover, critical issues were identified through thematic analysis, including concerns about small vessel representation, lack of coagulation, body donor preparation time and access, and limitations in body donor positioning. Addressing these aspects could give important added value to the simulation experience. While acknowledging the need for ongoing advancements to address these limitations, the study underscores the transformative potential of SimLife® in creating realistic, safe, and controlled environments for medical training and education. The overwhelmingly positive participant experiences and enthusiasm for repetition substantiate SimLife®'s efficacy and potential for further refinement in shaping the future of medical simulation technologies. Looking forward, addressing these limitations through innovations such as enhanced microvascular simulation, clotting models, and flexible donor configurations could significantly broaden SimLife®‘s applicability. These advancements have the potential to reshape medical training globally, particularly in underserved specialties requiring high precision, such as otorhinolaryngology and vascular surgery. Additionally, future research could explore tailoring questionnaires to specific specialties, with a focus on understanding how *pre-lauream* trainees perceive the differences between reperfused and non-reperfused body donors. Another important direction would be to analyze the detailed positive feedback given by specialties that found the simulation most beneficial compared to traditional surgical practice. SimLife® technology stands as not only a pivotal tool in medical training for its technological prowess but also as a beacon of ethical stewardship, ensuring that the legacy of body donors is honored. By continuing to integrate technological innovation with robust ethical frameworks, SimLife® has the potential to redefine the landscape of medical education and create a lasting impact on the next generation of healthcare professionals.

## Limitations of the study

The present study represents a significant advancement in medical education, offering a highly realistic platform for surgical and medical training. However, despite these positive attributes, the study faces several limitations. One primary limitation is the dependency on body donations, which can be unpredictable in terms of the number and characteristics of human bodies available. This limitation impacts the scope and diversity of simulations possible. Additionally, the limited time for conducting the simulations with human bodies is a significant constraint, as the body donors can only be used for a certain period before they become unsuitable for the simulations. Technical and organizational challenges also exist, including the complexity of preparing body donors for reperfusion and re-ventilation, as well as ensuring the proper functioning of SimLife® technology. These factors together limit the scale and variety of training scenarios that can be realistically simulated, which could affect the study’s generalizability and applicability to a broader range of medical and surgical training contexts. In addition to the limitations related to body donors, the study also faces constraints stemming from its design. The methodology employed may not fully replicate real-life clinical scenarios, potentially affecting the validity and applicability of the results. There is also a risk of bias due to the small sample size and the potential variability in human body conditions, which could impact the consistency and reliability of the findings. Furthermore, the study’s design might not adequately address all relevant variables in medical training, thus limiting the comprehensiveness of the conclusions that can be drawn from this research. Finally, mastering SimLife® technology also requires a learning curve itself. Therefore, it is necessary to perform more simulations to obtain better outcomes. With this future perspective in program, the Anatomy Center of the University of Bologna aims to become a national reference center for this groundbreaking technology.

## Data Availability

The raw data supporting the conclusions of this article will be made available by the authors, without undue reservation.
