# Supplementary material for: Body donor reperfusion and re-ventilation in medical training: an Italian study testing SimLife®
Source: Front Med (Lausanne). 2025 Jan 23;11:1488285. doi: 10.3389/fmed.2024.1488285 (PMC11799287; doi:10.3389/fmed.2024.1488285)
Supplement: Supplementary file 1 [file Data_Sheet_1.PDF]

Simedys Event Interview Scheme

Part 1

Question 1: Could you please indicate your gender? You're free to not answer.

Question 2: Could you please indicate your age? You're free to not answer.

Question 3: What is your medical specialty?

Question 4: What was your anatomical district of interest for this simulation?

Question 5: What intervention did you perform?

Question 6: Was the intervention you performed successful?

Part 2

Question 7: Evaluate the usefulness of SimLife Technology for the following scopes using a scale from 1 to 6, where 1 means "extremely useless", 2 means "useless", 3 means "somewhat useless", 4 means "somewhat useful", 5 means "useful", and 6 means "extremely useful":

- Research
- Pre-lauream Training
- Post-lauream Training
- Team building

Question 8: Evaluate how much did you overall enjoy this simulation, using a scale from 1 to 6, where 1 means "extremely dissatisfied", 2 means "dissatisfied", 3 means "somewhat dissatisfied", 4 means "somewhat satisfied", 5 means "satisfied", and 6 means "extremely satisfied".

Question 9: On a scale from 1 to 6, where 1 means "strongly disagree", 2 means "disagree", 3 means "somewhat disagree", 4 means "somewhat agree", 5 means "agree", and 6 means "strongly agree", how much do you agree with the following statement: I would like to repeat this experience.

Part 3

Question 10: Did you notice any critical issue during the simulation?

Question 11: Do you have any other additional comment?
