# Supplementary material for: Body donor reperfusion and re-ventilation in medical training: an Italian study testing SimLife®
Source: Front Med (Lausanne). 2025 Jan 23;11:1488285. doi: 10.3389/fmed.2024.1488285 (PMC11799287; doi:10.3389/fmed.2024.1488285)
Supplement: Supplementary file 2 [file Data_Sheet_2.PDF]

Supplementary Videos' Legends

Supplementary Video 1 – Shortcut of the galeo-pericranial microvascular free flap intervention. The video reports the creation of the anastomosis between the superficial temporal artery and the facial artery, performed at the mandible level.

Supplementary Video 2 – Shortcut of the knee medial and lateral ligament reconstruction intervention. The video displays the initial part of the intervention, when the ligaments are exposed. This shortcut allows the visualization of the avatar blood flow, that the surgeons must control, mimicking the intervention on live patients.
